# Functional and versatile superhydrophobic coatings via stoichiometric silanization

**DOI:** 10.1038/s41467-021-21219-y

**Published:** 2021-02-12

**Authors:** Lishen Zhang, Alvin G. Zhou, Brigitta R. Sun, Kennedy S. Chen, Hua-Zhong Yu

**Affiliations:** Department of Chemistry, Simon Fraser University, Burnaby, British Columbia V5A 1S6 Canada

**Keywords:** Wetting, Structural properties

## Abstract

Superhydrophobic coatings have tremendous potential for applications in different fields and have been achieved commonly by increasing nanoscale roughness and lowering surface tension. Limited by the availability of either ideal nano-structural templates or simple fabrication procedures, the search of superhydrophobic coatings that are easy to manufacture and are robust in real-life applications remains challenging for both academia and industry. Herein, we report an unconventional protocol based on a single-step, stoichiometrically controlled reaction of long-chain organosilanes with water, which creates micro- to nano-scale hierarchical siloxane aggregates dispersible in industrial solvents (as the coating mixture). Excellent superhydrophobicity (ultrahigh water contact angle >170° and ultralow sliding angle <1°) has been attained on solid materials of various compositions and dimensions, by simply dipping into or spraying with the coating mixture. It has been demonstrated that these complete waterproof coatings hold excellent properties in terms of cost, scalability, robustness, and particularly the capability of encapsulating other functional materials (e.g. luminescent dyes).

## Abstract (web summary)

Artificial superhydrophobic coatings that are simple to prepare and practical to use are sought after. Here, the authors create versatile, complete-waterproof coatings based on a single-step, stoichiometrically controlled reaction of organosilanes with water.

## Introduction

Superhydrophobicity is a commonly observed phenomenon in nature, which has been studied intensively and explored for applications in many different fields for decades^1,2^. In particular, surface coating techniques to attain ultimate waterproof capabilities have attracted both industrial and scientific interests^3^. To achieve superhydrophobicity, the general strategy is to create micro/nanostructures using materials of low surface tension^4,5^. In practice, most coating methods reported to date rely on either replicating pre-existing “rough” structures^6–13^ or creating roughness on existing materials via multi-step procedures^14–18^. Limited by the availability of ideal natural templates and simple fabrication procedures, a coating method that is facile, low-cost, scalable, and environmental-friendly is in great demand of all time^3^. To meet these application necessities, we herein explore an unconventional fabrication technique for superhydrophobic coatings based on a controlled, spontaneous reaction of organosilanes with a stoichiometric amount of water under ambient conditions.

For the coating fabrication, octadecyltrichlorosilane (OTS), a popular organosilane derivative, is the only reagent required other than water and a solvent (e.g., hexane). This mass-produced chemical is well-known for modifying the surface properties of various solid substrates by forming a compact and highly-oriented self-assembled monolayer (SAM) atop^19^. Its fluorine-free composition minimizes potential environmental and health hazards^20–22^. Conventionally, chlorosilane derivatives are reputed to vigorously react with even trace amounts of water and create large aggregates that are futile for obtaining high-quality monolayer coatings^23,24^. When adopting chlorosilanes for surface modification, a rigorously controlled humidity is generally required. However, for OTS, its C18 long alkyl chain sterically reduces the reaction rate when exposed to water or moisture. With the reaction being kinetically controllable, we were able to unorthodoxly harness the aggregation of OTS to create the desired surface coating upon reacting with a stoichiometric amount of water. In addition to its conventional application for forming SAMs on traditional substrates^19,25,26^, OTS has also been used to aid the preparation of superhydrophobic surfaces by forming such hydrophobic monolayers on “pre-made” nanostructured templates (Supplementary Table 1)^27–34^.

Different from those pioneering studies, the stoichiometric reaction between OTS and water explored herein not only creates the required hierarchical micro/nanostructures but also result in low surface tension. We are able to essentially achieve superhydrophobicity in a single step with OTS as the only precursor besides water and dilution solvent. This strategy is also conceptually different from conventional sol–gel processes, for which rather less-reactive organosilanes (e.g., tetraethoxysilane) were mixed with a bulk amount of water to create solid materials of controlled morphology and composition (e.g., thin films, grains, fibers, and porous gels)^35^.

## Results

### Superhydrophobic coatings via stoichiometric silanization

**Fig. 1:**
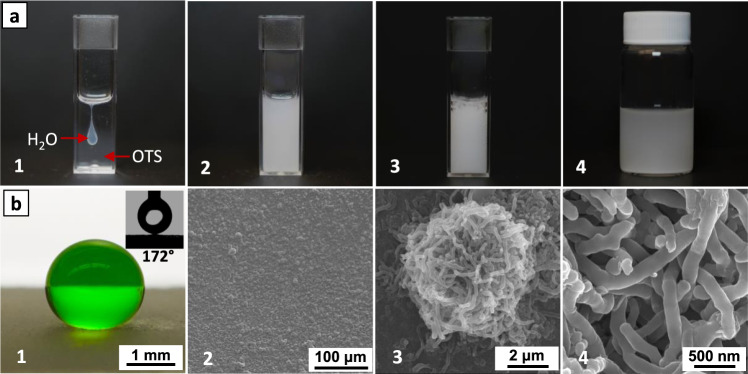
Preparation and characterization of the superhydrophobic coating. **a** Preparation of the coating solution that can be applied on various solid substrates: (1) 2.0 mL of pure OTS added with 40 μL of water; (2) upon mixing by vortex and sonication; (3) upon incubation for 2 h under ambient conditions; and (4) upon dilution with hexane (5% v/v OTS/hexane). **b** Morphological characterization of a microscope glass slide treated with the coating solution. The optical image on the left shows a water droplet (5.0 μL) on the surface (inset shows the measured water contact angle). The three SEM images of different magnificaitons show that the surface is covered with aggregated particles; these microparticles (2 to 20 μm) are consist of entangled nanofibers (width: 150–200 nm, length > 2 μm).

The coating mixture was prepared by directly reacting water with pure OTS, followed by dilution with hexane. As shown in Fig. 1a, the consecutive photos depicted a trial experiment of adding water to OTS with a mole ratio of 1:2, for example, 40 μL (2.2 mmole) of water is required for 2.0 mL of OTS (4.6 mmole), which is about 1/3 of the amount of water needed to complete the hydrolysis and condensation of OTS. The addition of water followed by immediate mechanical mixing, including vortex and sonication. The mixture was then diluted with hexane (5% v/v OTS/hexane) before being applied on a number of solid surfaces (vide infra). Remarkably, the reaction between water and OTS is rather mild with minimal volume increase and gas (HCl) release. We first investigated the modification of standard microscope glass slides, which is an intrinsically flat substrate. As shown in Fig. 1b, a 5.0 μL of water droplet stays as a near-perfect sphere on the surface and easily slide off (Supplementary Movie 1). The water contact angle of the modified glass was measured to be 172 ± 1°, with an ultralow sliding angle of 0.7 ± 0.2°. The surface was also tested dynamically with a 5.0 μL water droplet at a speed of 1 m s^−1^ (momentum energy of 2.5 μJ), and the droplet bounced off the surface freely without pinning onto it (Supplementary Movie 2). As captured by a high-speed camera (Supplementary Movie 3), a water droplet freely jumped from one side to the other side of the superhydrophobic glass slide. Apparently, both the ultrahigh water contact angle and ultralow sliding angle (minimum wetting hysteresis) warrant the excellent water-repellency (non-sticky property) of the modified glass surface.

After modification, the glass slide is covered with a layer of uniform, micro-size particles with diameters ranging from 2 to 20 μm (Fig. 1b). The high-resolution scanning electron microscope (SEM) illustrated that these microparticles are, in fact, formed from entangled nanofibers (150–200 nm in diameter and 2–10 μm in length), which beautifully mimics the micro-to-nanoscale hierarchical morphology on lotus leaves^3^. More impressively, the seminal hierarchical model proposed previously by Feng et al.^7^ can be adapted for simulating such a superhydrophobicity:

1 $$\cos \theta _{app} = f_s\left( {L/l} \right)^{D - 2}\cos \theta - f_v$$

The value of *D* (fractal dimension) in three-dimensional space is 2.2618^7^. For the modified glass surface (Fig. 1b), the average value of *f*_*s*_ was estimated to be 0.2 (*f*_*v*_ = 0.8), *L* is 10 μm and *l* being 175 nm. The calculated water contact angle is 171°, which matches the experimental determination very well (172 ± 1°). This result affirms that the superhydrophobicity of our modified surfaces should be attributed to the lotus-leaf like micro-to-nanoscale hierarchical roughness.

**Fig. 2:**
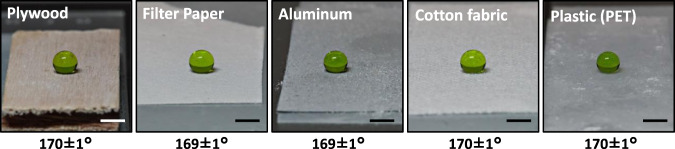
Creation of superhydrophobicity on a diverse set of solid materials. Water droplets (7.0 μL) on different substrates that were treated with the coating solution. The value below each image is the corresponding water contact angle; the sliding angles of all modified samples are within 0.5–1.0°. All scale bars in the pictures are 2 mm.

Besides the glass surface discussed above, the coating solution can be applied to many other different materials regardless of roughness, composition, and rigidity. Particularly, we have demonstrated the above coating method on (1) paper (laboratory filter paper), (2) fabric (100% cotton shirting fabric) (3) wood (maple plywood), (4) metal (aluminum thin sheet), and (5) plastics (polyethylene terephthalate) as immediate examples. As shown in Fig. 2, all solid substrates that were treated with the coating solution resulted in high water contact angles (168–171°) and very low sliding angles (0.5–1.0°). The aforementioned micro-to-nanoscale hierarchical structure has been also confirmed on other substrates (e.g., porous filter paper, Supplementary Fig. 1). Their superior water-repellency property has been further illustrated with a demonstration clip (Supplementary Movie 4) to show how water droplets bounce off these treated surfaces.

### Optimized reaction protocol and mechanistic investigation

**Fig. 3:**
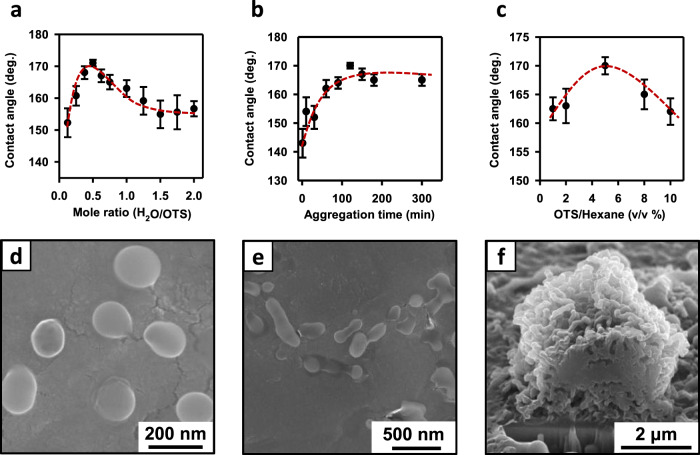
Optimized fabrication and mechanistic investigation. **a**–**c** show the obtained water contact angle on modified glass slides as a function of the mole ratio (H_2_O/OTS), aggregation time, and dilution factor of the aggregated OTS in hexane, respectively. The errors represent the standard deviations from at least three independent experiments. **d** and **e** are SEM images of the hierarchical aggregates at different reaction stages. **f** shows the cross-section of a particle “anchored” on the substrate surface (cut with FIB).

**Fig. 4:**
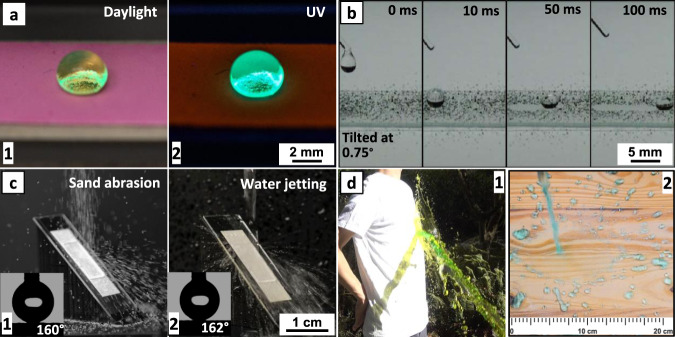
Encapsulation capability and real-life applicability of the superhydrophobic coating. **a** A drop of dye (pyranine) solution on a superhydrophobic filter paper surface encapsulated with a luminescent chromophore (Rhodamine B) under daylight (**a**-1) and UV lamp illumination (**a**-2); **b** demonstration of the self-cleaning property on a treated glass slide; **c** mechanical stability test of modified glass slides with sand abrasion (**c**-1) and water jetting (**c**-2) experiments; the insets show water contact angles measured on the surface after the tests; **d** side view of water splashed off from the treated, superhydrophobic cotton T-shirt (**d**-1); water splashing test on a large piece of pine wood modified with the spray method (**d**-2).

Besides the superior water-repellent property, the nature of such an unconventional alkylsilane/water reaction is also intriguing, which correlates with the optimization of the reaction conditions. As shown in Fig. 3a, the key factor is the amount of water added to the OTS, i.e., at a molar ratio of 1 : 2 (water : OTS) the best performance was achieved. Either less or more water would lead to a decrease in the water contact angle of the treated surface. As mentioned above, this stoichiometrically controlled hydrolysis and condensation of OTS is conceptually different from either the monolayer formation with long-chain alkylsilanes (which is performed under a strictly controlled humidity) or the conventional sol-gel process (which mixes less-reactive organosilanes with a bulk amount of water)^19,36^. Although the molar ratio between the two reactants is 1:2, the volume percent of water in the mixture is only ~2%. After applying the mechanical dispersion, such a small amount of water mixes well with OTS and quickly decreases to submicron-to-nanometer size droplets^37^. This leads to the formation of a stable and uniform water–OTS emulsion with the hydrophilic end of OTS molecules orienting towards water droplets and the hydrophobic chain facing outward (Supplementary Fig. 2a). The continued hydrolysis and subsequent condensation of OTS consumes water and generates HCl, which creates an acidic condition for further catalyzing the reaction^36,38^. Consequently, spherical nanoparticles (100–200 nm) are formed with the surface covered with alkyl chains (Fig. 3d, Supplementary Fig. 3a and Fig. 4). Next, nanoparticles tend to form head-to-head linear fibers (Fig. 3e and Supplementary Fig. 3b), because of the energy barriers to encounter during aggregation^39^. Then, linear fibers further aggregate and eventually form micro-size particles (Fig. 3f, Supplementary Fig. 2b and Fig. 3c). This silanization reaction can last for ~6 h (Supplementary Fig. 5), although the highest water contact angle (172°) is achieved after 2 h (Fig. 3b). The continued variation of the resulted water contact angles after this point indicates that the reaction was not complete, i.e., reactive sites (-Si-OH or -Si-Cl) on the particles still exist. Therefore, micro-size aggregates can covalently bond to the substrates providing that hydroxyl groups are present^38^, i.e., the particles would bond to the surface and form hierarchical structures. In Fig. 3f, we have shown the “FIB cut” cross-section of a particle that is “anchored” on the substrate surface (also shown in Supplementary Fig. 3d and Supplementary Fig. 6).

In order to examine the composition of the superhydrophobic coatings, both Fourier transform infrared (FTIR) spectrum and X-ray photoelectron spectrum (XPS) were acquired on modified aluminum and glass samples, respectively. The band at 1003 cm^−1^ in the FTIR spectrum is due to the bending vibration of Si-O-Al, which verifies the formation of chemical bonding on the surface (Supplementary Fig. 7)^40^. For the XPS results, the C/O/Si atomic ratio changed from 7.41/66.43/26.16% to 86.37/9.10/4.53%. The significant increase in the C*1s* peak intensity after the modification confirms the existence of OTS aggregates on the surface (Supplementary Fig. 8). The hypothesis and condensation of OTS for the formation of the hierarchic structure was further confirmed by adding HAuCl_4_ in water when preparing the coating mixture to trace the entire process. We were able to detect the existence of gold elements in the emulsion and therefore verified the presence of water (Supplementary Fig. 6a and b) in the fibers formed from siloxane particles. Meanwhile, in between particles the surface was covered with small particles (Supplementary Fig. 6c, d). It should be emphasized that the entire reaction was performed in a controlled manner, which complements the conventional utilization of organosilanes for sol-gel reaction or surface modification as mentioned above^19,35^. The other important fabrication step is the dilution of the stock solution (Fig. 1a-4) by adding common organic solvents (hexane or mineral spirit) of preference. As shown in Fig. 3c the optimal concentration was determined to be 5.0% (v/v). This relatively low concentration enables the potential for large-scale fabrication of the coating.

### Encapsulation capability and real-life applicability

As mentioned above, an inspiring phenomenon is that gold nanoparticles can be encapsulated in the coating (Supplementary Fig. 6). To further test and visualize this capability, water-soluble fluorescent dye (Rhodamine B) was tested as a trial luminescent chromophore. The ring-open structure of Rhodamine B makes the molecule polar and soluble in water but not in organic solvents. The solution of Rhodamine B (30 mM), instead of pure water, was added to the OTS in the procedure mentioned above (Fig. 1a) and the coating mixture was then applied on laboratory filter paper. The resulted surface displayed a pink color under ambient light; the color remains after washing with water and organic solvents, which indicating that the dye molecules are not physically adsorbed on the surface. As shown in Fig. 4a, a water droplet (dyed with another water-soluble fluorescent dye, pyranine) sits perfectly atop, which confirms the unperturbed superhydrophobicity (Fig. 4a, left photo). It is more remarkable that under UV light (*λ* = 254 nm), this superhydrophobic filter paper displayed bright red emission, whereas strong green fluorescence was observed from the water droplet (right picture of Fig. 4a and Supplementary Movie 5). The reflections from air bubbles trapped under the water droplet validate the Cassie-Baxter wetting state^41^. The fact that the encapsulation of chromophores does not influence either ultrahigh water contact angle or the sliding angle, warrants broader application potentials such as designing colorful and luminescent waterproof coatings.

The other intriguing property of superhydrophobic surfaces is the self-cleaning capability, which can be adapted to various daily life scenarios such as building construction, clothing, and machining materials. In Fig. 4b (also Supplementary Movie 6), we have demonstrated with a series of photos taken at different time frames that a water droplet readily rolls off a slightly tilted (~0.75°) glass slide prepared above and carries away the dust (MnO particles) from the surface.

In addition to the application versatility, mechanical stability is considered a vital criterion for surface coatings^42,43^. To apply the coating in daily life, superhydrophobic surfaces are desired to survive under harsh weather conditions. The mechanical stability of surface coatings was typically tested by sand abrasion and water jetting experiments. As shown in Fig. 4c and Supplementary Movie 7, the modified superhydrophobic glass substrate remained superhydrophobic (>160°) after sand abrasion or water jetting for 10 min. It was also confirmed that water immersing for 3 days and tissue wiping for 20 times did not change the surface hydrophobicity significantly. For a comparison with the best performing superhydrophobic coatings reported recently^9,42^, we have conducted the “standard” abrasion test, in which the modified glass substrate was pressed onto a piece of silicon carbide sandpaper (Grit No. 400) under the pressure of 2.5 kPa, and then abraded for a distance of 50 cm (Supplementary Fig. 9 and Movie 8). The glass slide retained a high contact angle (161 ± 2°) and a low sliding angle (~1°) after the abrasion test. Such an exceptional anti-abrasion property can be explained by the fact that the siloxane aggregates are covalently bonded to the surface. As shown in Fig. 3f and Supplementary Fig. 10, the SEM image with a FIB cut shows that the hierarchical particles and the solid substrate are in fact “merged” together owing to the continued formation of siloxane compounds (as discussed above). The chemical bonding provides higher stability compared with physical adsorption or deposition, leading to the strengthened robustness of the superhydrophobic coating^38,44^. In addition, the anti-abrasion property could be also attributed to the fiber-entangled porous structure of the micro-size particles. As illustrated with SEM imaging (Supplementary Fig. 11), the abrasion test indeed partially removes entangled nanofibers at the top surface, the substrate is still consisting of “similar” hierarchical nano/microstructures. That is, the remaining structure resembled the original surface prior to abrasion (Supplementary Fig. 12). Peng et al.^8^ previously achieved such robustness (anti-abrasion property) by creating a self-healing, soft, and self-similar morphology; we demonstrated that the combination of chemical bonding and self-similar structural strategy can achieve the superior anti-abrasion property as well. Such mechanically robust coating is potentially useful for building and ship construction, as well as outdoor gears, which typically sustains harsh conditions from wind, rain, or wear and tear.

As the present protocol was found effective on a variety of substrates in small batch, we further evaluated the industrial scalability of the coating method, which is a key factor in practice^3^. To mimic the mass production of such coating in clothing industry, a regular cotton T-shirt treated with the coating solution has exhibited an excellent water repellency (Fig. 4d and Supplementary Movie 9). Water (with green dye) readily bounced off the surface, leaving the T-shirt clean and dry. It is noteworthy that the superhydrophobic fabrics remained good air permittivity (Supplementary Fig. 13), augments the potential of making commercial waterproof, breathable coating products from porous and flexible materials.

The subsequent test is to compare the results from two alternative application methods: dipping the substrate into the coating solution and spraying the coating solution onto the substrate. For any same type of solid materials, we did not observe significant differences in the wetting performance when different methods were used to prepare the samples. Nonetheless, almost all commercial products today are for spray coating, as it is easy-to-use with the coating solution contained in a spray bottle. Upon testing different organic solvents (Supplementary Fig. 14) to prepare the spray solution, we discovered that the wetting performance is better by using solvent with lower polarity. Aligning to the aim of industrial applications, a widely used spray coating solvent, mineral spirit, was employed during the dilution step (Figs. 1a-4). The performance of the spray coated samples were compared with coating products from several commercial brands, namely Wood^TM^, Grangers^®^, Kiwi^®^, Nikwax^®^, Neverwet^TM^, and Scotchgard^TM^, on glass and cotton fabrics. The surfaces treated with our coating solution (sprayed and air dried for 2 h) showed superior waterproof properties, for which we have achieved superhydrophobicity on both types of material (162°−165°). Meanwhile, other coating products tested merely reached the level of hydrophobic (90°–120°) (Supplementary Table 2). The coating solution was also sprayed on a large piece of pine wood to further demonstrate the scale-up capability; as shown in Fig. 4d (right) water would not stick to the treated wood surface and runs off readily.

The last but not the least sets of application requirements for the coating are the aging effect, anti-icing property, and cost-effectiveness. We have shown that after exposure to ambient conditions for 18 months, the coating applied on a number of surfaces demonstrated no sign of degradation. Particularly, treated cotton fabric and plywood samples tested after various periods of storage time remain superhydrophobic consistently (Supplementary Fig. 15).

More importantly, the cost for producing large waterproof surfaces with the present coating method is inexpensive since there are no intricate instrumentation and expensive materials involved in the production process. It was estimated that >100 kg of the coating solution can be produced with a cost of ~300 US$ and the production can be carried out per operator on a daily basis under ambient conditions. Furthermore, this protocol is based on a rather simple, and mild hydrolysis/condensation reactions of organosilanes. The only byproduct is HCl, which can be recycled for producing the precursor^45^. Therefore, in principle, there is minimal environmental impact, and the simple synthesis and instrument-free production warrant large-scale industrial production.

## Discussion

Comparing with other state-of-the-art coating methods, our method uses only OTS and water to create hierarchical micro/nanostructure template with low surface tension in a single step, and demonstrates superior wettability and anti-abrasion property (Supplementary Table 3)^6,8–10,43,46–53^. In comparison, most other strategies relied on the modification of “pre-made” rough structures with low surface tension coatings. Therefore, with such a unique stoichiometric silanization approach the fabrication procedure is largely simplified, for which the chemical reagents are readily accessible, and the preparation is scalable for industrial applications.

We also tested several other alkyltrichlorosilanes to compare the resulting wetting property of treated samples with that of OTS. It was found that alkyltrichlorosilanes with slightly shorter chains, namely hexadecyltrichlorosilane (HTS, C16) and dodecyltrichlorosilane (DTS, C12), can be adapted with the same protocol developed for OTS to modify various solid substrates. The much shorter ones, such as methyltrichlorosilane (MTS), are much more reactive and cannot be used with the exact same protocol. A summary of the resulting wettability for filter paper modified with alkyltrichlorosilanes of varying chain lengths is presented in Supplementary Fig. 16. Although the stoichiometric silanization with HTS (C16) and DTS (C12) can indeed modify filter papers to be hydrophobic (137 ± 5° and 125 ± 6°) but does not achieve the same level of superhydrophobicity (as OTS does). In addition, the modification with other types of organosilanes (e.g., trichloro(1H,1H,2H,2H-perfluorooctyl)silane, trimethoxyoctadecylsilane) was explored as well, however, their hydrolysis reaction is too slow, i.e., they cannot effectively form micelles in the presence of the stochiometric amount of water.

Another aspect of such a nanostructured, superhydrophobic surface is the potential anti-icing property. Our initial tests indicated that ice form with the same amount of water (30 μL) has a smaller contact area with the modified superhydrophobic substrate (Supplementary Fig. 17), and the measured detachment force (contact area normalized) decreased ~30%, from 895 ± 69 kN to 636 ± 36 kN. Although the present coating indeed exhibited anti-icing property, it is not yet comparable with other specially prepared systems^54,55^. More comprehensive investigations of the coating morphology using other microscopy techniques (e.g., atomic force microscopy) and of expanded functionalities (including anti-icing property) are certainly warranted, yet are beyond the scope and focus of this report (i.e., exploration of the stoichiometric silanization to create superhydrophobic coating).

In summary, the developed superhydrophobic coating technique, based on an unconventional, stoichiometrically controlled aggregation of organosilanes, promises a versatile and practical method for surface modification at industrial scales for real-life applications. This protocol eliminates many limitations of today’s waterproof coating methods, such as expensive materials and time-consuming preparation procedures. Moreover, the versatility of our coating technology enables the possibility of utilizing the surface coating over many different solid substrates, despite their varied surface morphology and sample dimension. The created micro-to-nanoscale hierarchical structures can also be extended as templates for other micro-/nano-fabrications, to be performed on benchtop under ambient conditions.

## Methods

### Preparation of coating solution and treated surfaces

For the initial preparation of the coating solution as described in Fig. 1a, 20 μL water was added to 1.0 mL pure OTS in a 1.7 mL microcentrifuge tube. The tube was then caped and immediately put on a vortex mixer at 3200 rpm for 10 s, followed by sonication in an Ultrasonic cleaner for 10 s (uncapped) and another round of vortex mixing for 10 s (capped). Immediately after, 500 μL of the resulted emulsion was transferred to a 20 mL scintillation vial (with cap on but not airtight). After 2 h, 10 mL hexane was added to the vial and mixed by shaking before use.

For the surface modification, all solid substrates were cut into small pieces (1 × 3 cm^2^) and immersed in the coating solution prepared above for overnight. The treated sample was then removed from the solution and washed three times with hexane and then dried in air. For the spray coating experiments, all samples were used in their original form without any pretreatments except for washing with water and air drying. An exemption is for glass slides, for which an additional cleaning step was performed with an UV–Ozone cleaner for 30 min.

For the gold “tracking” and chromophore encapsulation experiments, 60 mg chloroauric acid was dissolved in 100 μL water or 15 mg of rhodamine B was dissolved in 1.0 mL water; the prepared solution was then used in place of water in the above steps to prepare the coating solution.

For the large-scale preparation and for spray coating experiments, 200 μL deionized water was added to 10 mL of OTS in a glass vial. Followed by the same preparation procedures as described above. After 2-h incubation of the mixture, the solution was diluted with 200 mL of mineral spirit. Then the solution was transferred to a plastic spray bottle. The spraying was performed above the sample at a 30° angle toward the sample to be treated.

### Characterization and instrumentation

Photos and normal speed videos were captured with a Sony mirrorless digital camera (Alpha a7RII, Japan) with a Canon macro lens (EF 100 mm f/2.8 L IS USM, Japan). The slow-motion video was captured with a high-speed camera (Promon U750) from AOS Technologies AG (Baden, Switzerland).

Water contact angles were measured with an optical goniometer (AST VCA system, Billerica, MA). A 1.0 μL droplet was held with a syringe needle, slowly moved down to contact the sample surface. At least three samples prepared under the same condition were tested; for each sample, five different regions were examined.

The morphology of the treated samples was imaged with a FEI Nova NanoSEM 430 system (FEI Company, Hillsboro, OR). The substrates were first sputtered with Ir (5 nm) with a Leica EM ACE600 (Wetzlar, Germany) deposition chamber to improve the conductivity. The cross-section view of the samples was obtained either using a FEI Strata DualBeam DB235 (FEI Company, Hillsboro, OR) or a FEI Helios NanoLab 650 SEM/FIB System. The substrates were coated with carbon (15 nm) in this case. During the imaging, the samples were tilted for 52° following by a gallium ion beam (30 pA) cutting for 15 min. Transmission electron microscopy (TEM) was carried out with a FEI Tecnai Osiris S/TEM (FEI Company, Hillsboro, OR) at 200 kV. The nanospheres were prepared as described above, then dissolved in hexane and transferred to a Cu-grid for imaging.

Elemental analysis was acquired with an EDAX detector installed on the FEI Strata DualBeam DB235 system. The element mapping was performed using the EDAX detector installed on the FEI Helios NanoLab 650 SEM/FIB System (with the energy at 8 kV). XPS data were obtained on an Axis Ultra DLD spectrometer (Kratos Analytical, Manchester, UK), with a monochromatic aluminum source (Al Kα 1486.6 eV) at a power of 150 W (10 mA/15 kV). The FTIR spectrum was acquired with on a Perkin Elmer Spectrum Two Spectrometer with an ATR attachment. The dynamic light scattering data were obtained using a Zetasizer Nano ZS system (model ZEN 3600) from Malvern Instruments, UK. The viscosity of OTS (14.674 mPa s^−1^ at 20 °C) was determined with a μVisc viscometer (RheoSense, Inc., San Ramon, CA); its refractive index (1.5122 at 532 nm) was determined with a Metricon refractometer (Model 2010/M, Pennington, NJ).

## Supplementary information

Supplementary Information

Supplementary Movie 1

Supplementary Movie 2

Supplementary Movie 3

Supplementary Movie 4

Supplementary Movie 5

Supplementary Movie 6

Supplementary Movie 7

Supplementary Movie 8

Supplementary Movie 9

Description of Additional Supplementary Files

## Data Availability

All relevant data are available from the authors upon request.
