# Supplementary material for: Functional and versatile superhydrophobic coatings via stoichiometric silanization
Source: Nat Commun. 2021 Feb 12;12:982. doi: 10.1038/s41467-021-21219-y (PMC7881188; doi:10.1038/s41467-021-21219-y)
Supplement: Supplementary file 1 — Supplementary Information [file 41467_2021_21219_MOESM1_ESM.pdf]

Supplementary Information

Functional and versatile superhydrophobic coatings via stoichiometric silanization

Lishen Zhang, Alvin G. Zhou, Brigitta R. Sun, Kennedy S. Chen, and Hua-Zhong Yu*
Department of Chemistry, Simon Fraser University, Burnaby, British Columbia V5A 1S6, Canada
Correspondence to: hogan_yu@sfu.ca

This PDF file includes:

Materials and Reagents
Supplementary Figures (Fig. 1 to 17)
Supplementary Tables (Table 1 to 3)

Other Supplementary Materials for this paper include:

Supplementary Movie 1 to 9 (supplied as independent files)

Materials and Reagents:

Octadecyltrichlorosilane (OTS, ≥90%), hexadecyltrichlorosilane (HTS, 95%), dodecyltrichlorosilane (DTS, 98%), methyltrichlorosilane (MTS, 99%), rhodamine B (≥95%; HPLC), 8-hydroxypyrene-1, 3, 6,-trisulfonic acid trisodium salt (pyranine, ≥99%), MnO powder (60 mesh, 99%) and chloroauric acid (≥99.9%, trace metals basis) were ordered from Sigma Aldrich (St. Louis, MO). Mineral spirit (Recochem Inc., Vancouver, BC) was purchased from Canadian Tire. Hexane (ACS reagent grade, ACP Chemical Inc. Montreal, QC). Sand standard (Ottawa, particle size 30-100 mesh, Anachemia, Montreal, QC) was purchased locally. Deionized water (>18.2 MΩ cm) was produced with a Barnstead EasyPure UV/UF compact water system (Model No. D8611, Dubuque, IA).

Supplementary Figures:

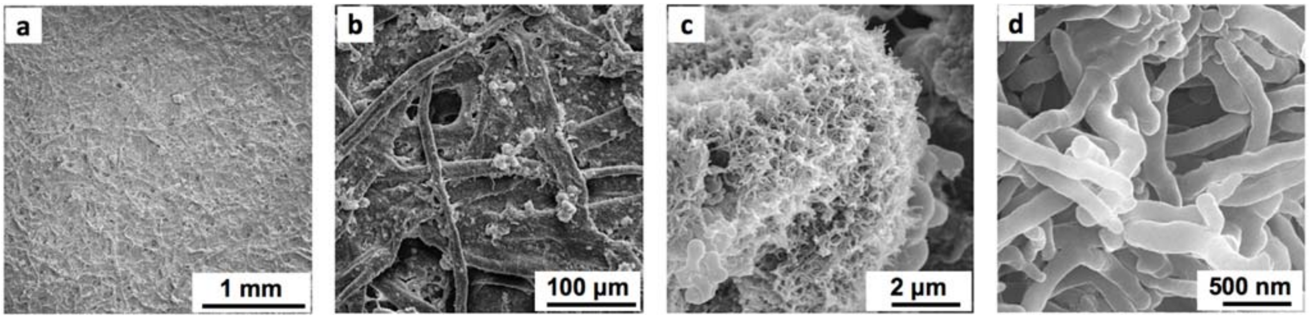

Supplementary Fig. 1 Morphology of the coated superhydrophobic filter paper. The sample (WhatmanTM grade 1 filter paper) was prepared according to the procedures described in the Method section. From **a** to **d** are SEM images of different magnifications. The cellulose microfibers are coated with microparticles that are consisted of entangled nanofibers, which was also observed on the treated glass slides (Fig. 1b in the main text).

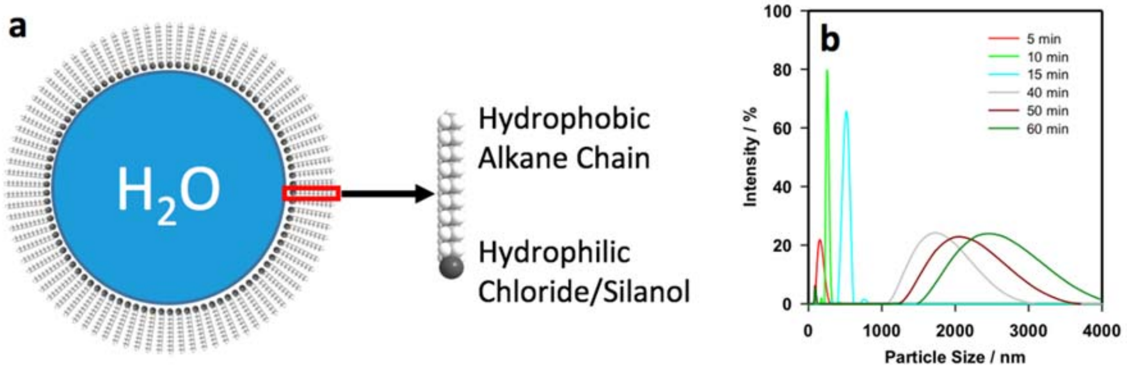

Supplementary Fig. 2 Analysis of the initial mixing and reaction between water and OTS. a Schematic view of a micelle of OTS formed around a water microdroplet. **b** The aggregated particle size vs. incubation time based on the DLS measurements. The average particle size increases with the incubation time, which reaches 2-3 μm in about an hour.

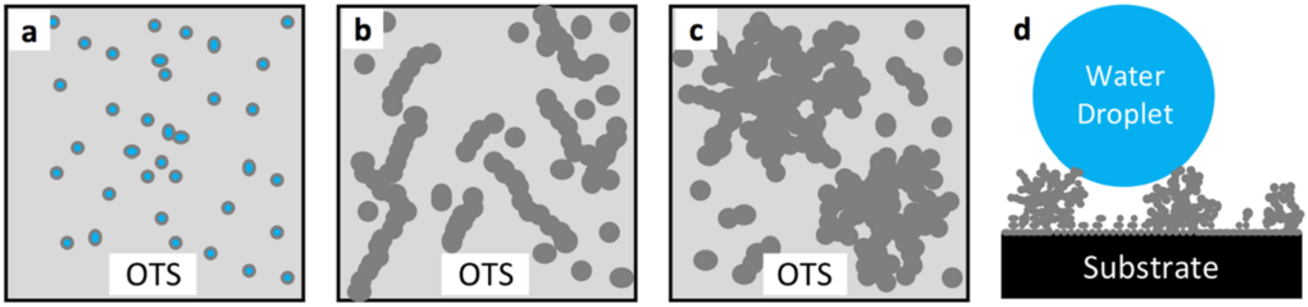

Supplementary Fig. 3 Schematic illustration of the formation of superhydrophobic coating. a Formation of micelles (blue circles) when introducing water into OTS (grey matrix). **b** Formation of nanoparticles (dark grey) resulted from the facile hydrolysis and condensation of OTS (light grey matrix); the nanoparticles in turn form head-to-head linear fibers. **c** Fibers aggregate and form the micro-size particles (dark grey). **d** Side-view of the coated surface with a water droplet on top.

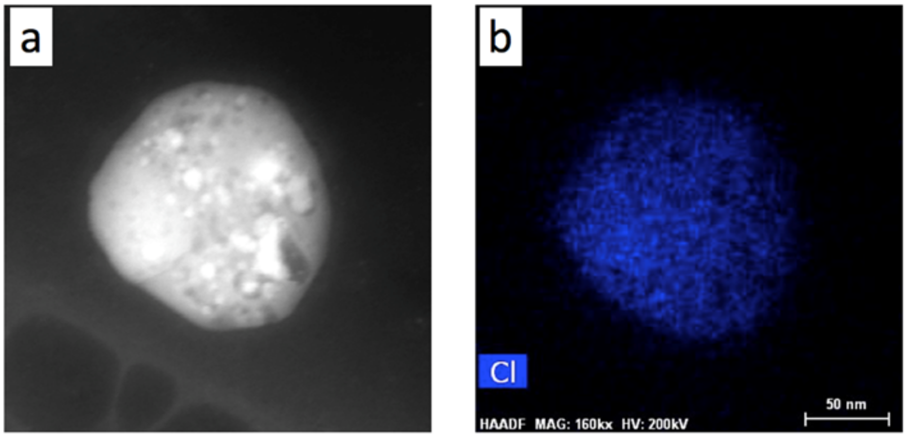

Supplementary Fig. 4 Analysis of the particle formation in the coating mixture. a TEM image of a nanosphere formed after the mixing step. **b** Elemental mapping of Cl on the surface of the same nanosphere. The TEM image was consistent in size and morphology with the observation reported in Fig. 3d of the main text. The element mapping analysis demonstrated that Cl still exists on the nanosphere surface after the initial mixing, indicating that the nanospheres remain reactive for forming head-to-head linear fibers subsequently.

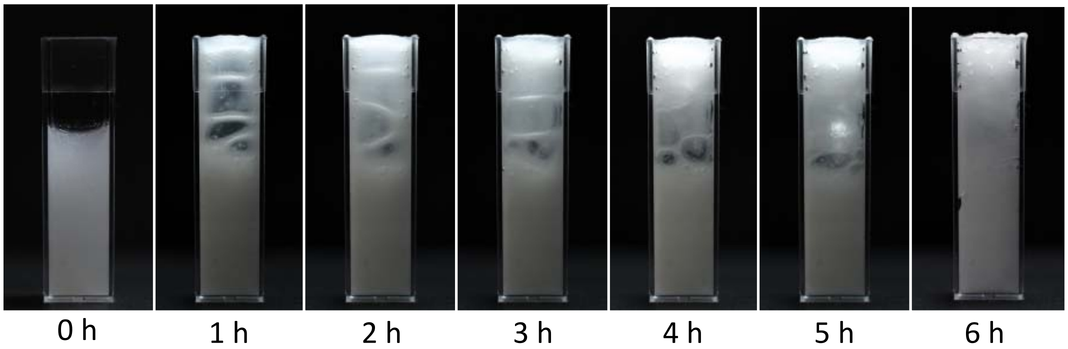

Supplementary Fig. 5 Progression of OTS/water reaction. For better visualization, a 3 mL reaction mixture prepared according to the procedure described in the Method section was transferred to a plastic cuvette. The reaction was initially fast, particularly for the first hour, with a noticeable amount of gas generated. It slowed down after 2 h. At about 6 h, the reaction was completed, and the product turned into a gel that was no longer dispersible in a solvent.

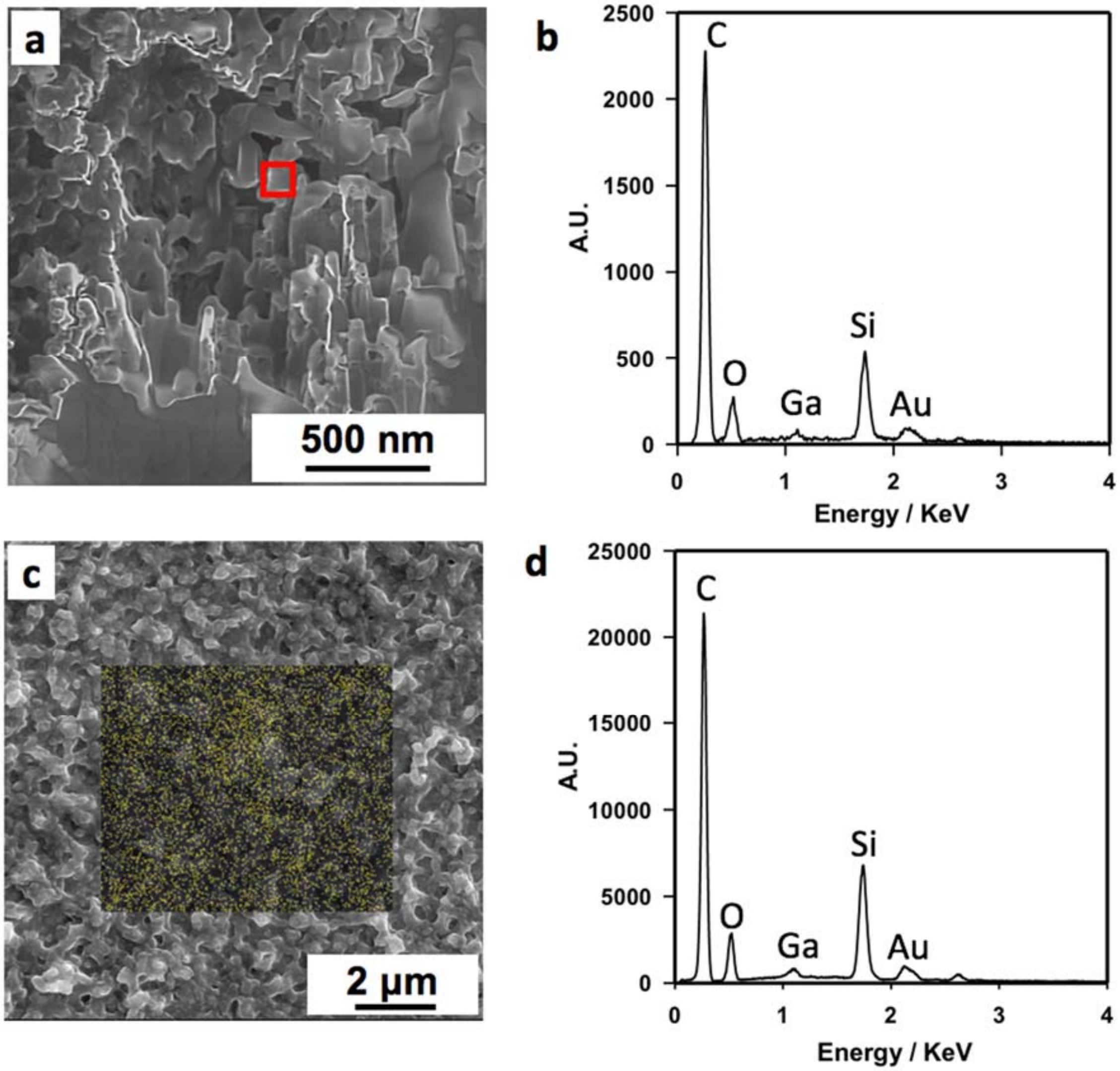

Supplementary Fig. 6 Investigating the formation of nano-aggregates via "gold tracking". a Side view of a FIB-cut particle on the treated glass surface. The EDX data **b** shows the elemental composition of red square in **a.** As gold salt is soluble in water and insoluble in organic solvents (hexane and OTS), the presence of Au in every nanofiber, indicates that these fibers are formed from the sols. **c** SEM image of the surface in between microparticles (inset is the overlay of gold mapping with surface morphology). **d** Elemental composition with the gold peak clearly shown.

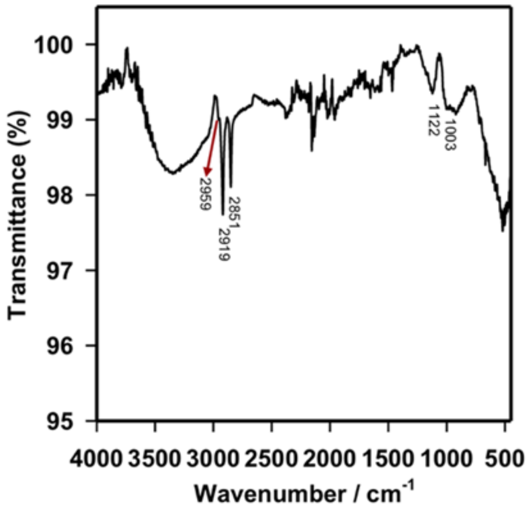

Supplementary Fig. 7 FT-IR spectrum of a treated aluminum plate. The peak at 1003 cm^{-1} is attributed to the asymmetric stretch of Si-O-Al groups, which confirms the formation of chemical bonds between the coating layer and the aluminum substrate. The asymmetric stretch of Si-O-Si group was located at 1122 cm^{-1}. Peaks at 2851 cm^{-1} and 2919 cm^{-1} were assigned to the symmetric and asymmetric stretching modes of $–CH_2$ groups from the long chain alkyl group of OTS, respectively. The relatively weaker peak at 2959 cm^{-1} can be attributed to the asymmetric stretching of the $–CH_3$ groups (of OTS) on the coating surface.

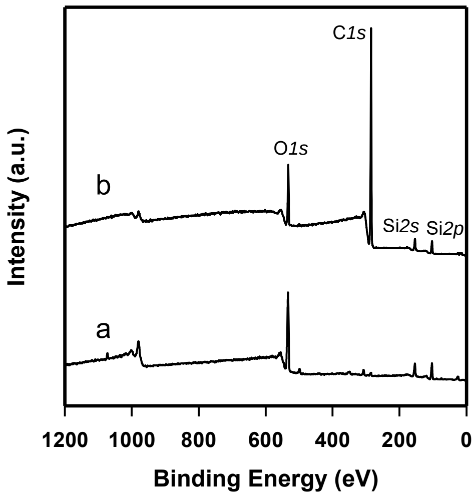

Supplementary Fig. 8 XPS spectra of a treated glass slide. XPS spectra before (**a**) and after (**b**) applying the superhydrophobic coating. Analysis of the spectra showed that the C/O/Si atomic ratio changed from 7.41%/66.43%/26.16% to 86.37%/9.10%/4.53%. The significant increase in the C*1s* peak intensity confirmed the presence of OTS and its aggregates on the glass surface.

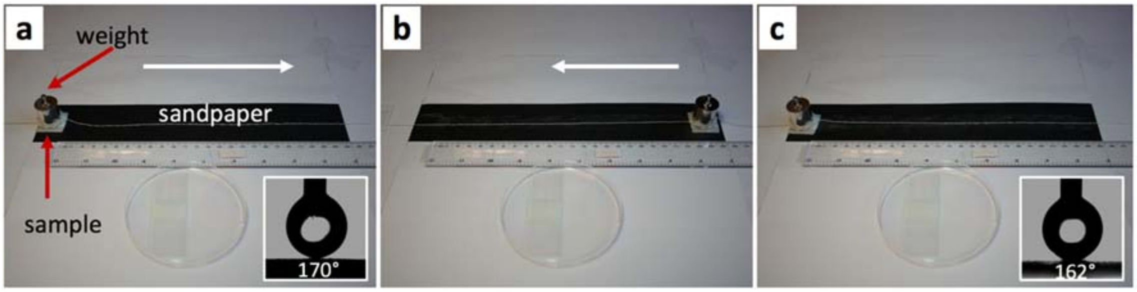

Supplementary Fig. 9 Abrasion test of treated filter paper. A small piece of the treated paper (1 × 2 cm^2) was affixed at the bottom of a weight (50 g), which was then dragged on the surface of sandpaper (Grit No. 400). From **a** to **c** shows one cycle of abrasion (50 cm); this method was adapted from Ref. 9. Water contact angles were measured before (inset in **a**) and after (inset in **c**) the test.

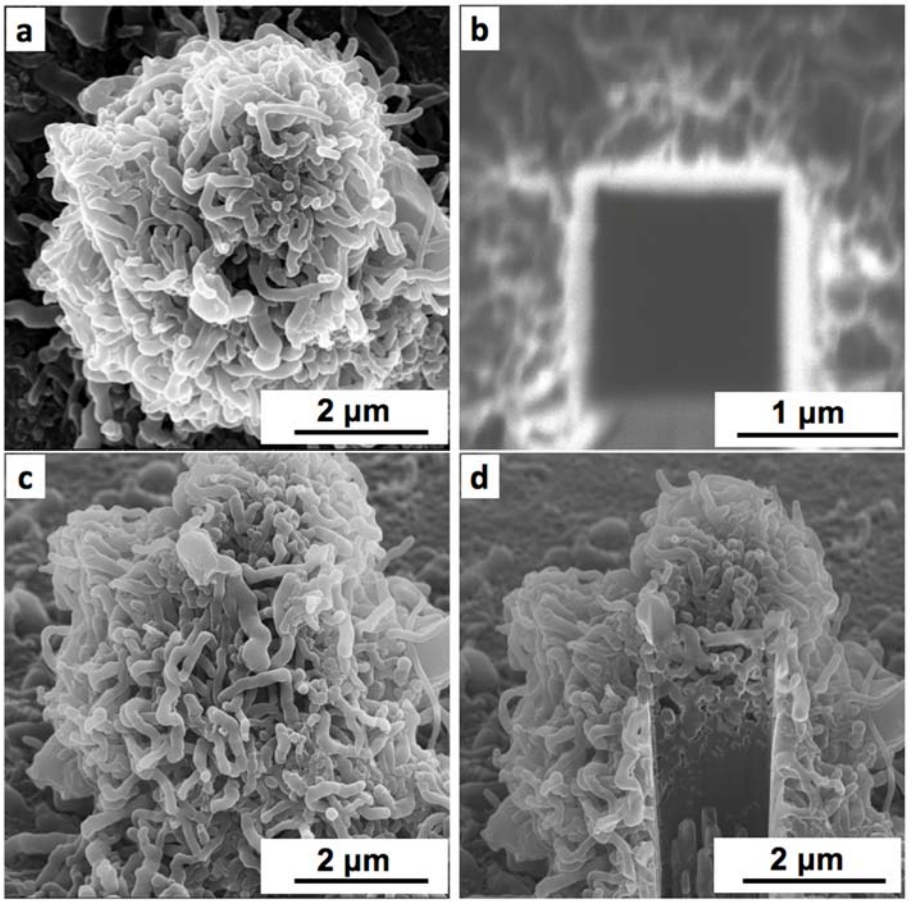

Supplementary Fig. 10 SEM characterization of hierarchical particles of a superhydrophobic coating. Top (**a** and **b**) and side view (**c** and **d**, tilted for 52°) of the micro-size particles on treated glass surface before (**a** and **c**) and after (**b** and **d**) FIB cutting. There is no discernible boundary between the particle and surface.

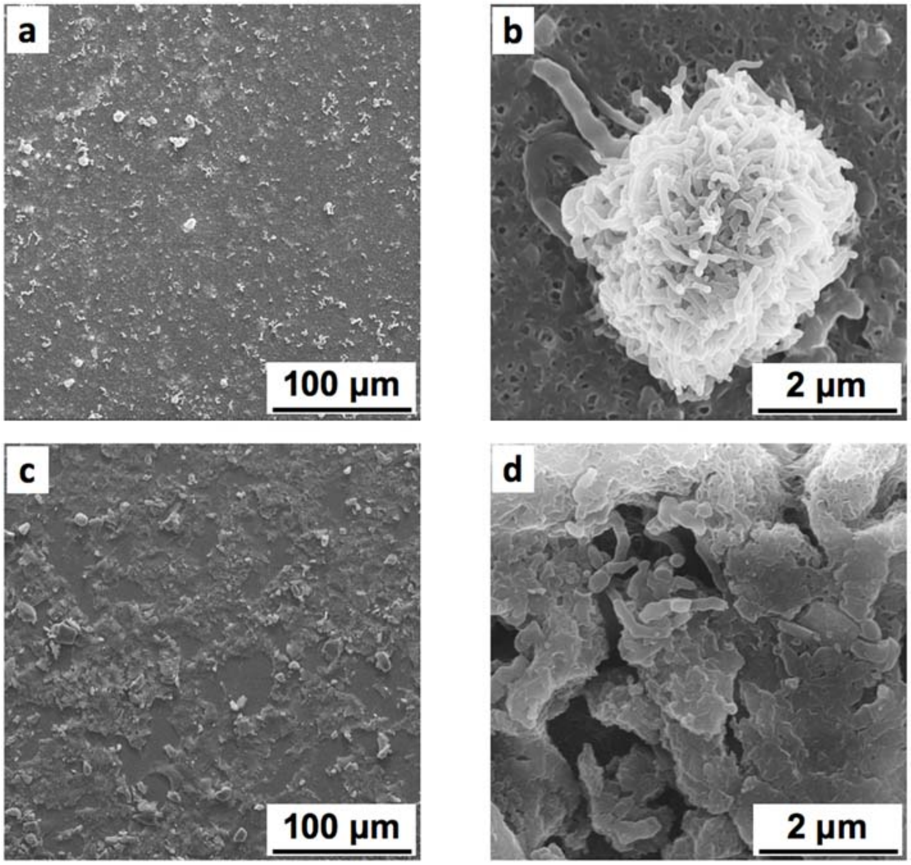

Supplementary Fig. 11 Morphology comparison before and after the abrasion test. SEM images of the superhydrophobic glass surface before (**a** and **b**) and after (**c** and **d**) sand abrasion, with **b** and **d** being the higher magnification images. Comparing the two sets of images, the abrasion process has partially removed the top layer of the original coating; the remaining surface, however, consists in a similar hierarchical nano/microstructure.

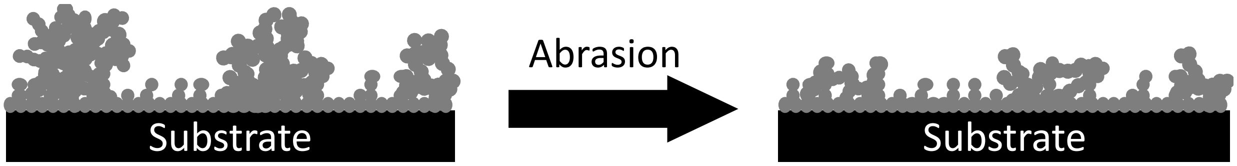

Supplementary Fig. 12 Schematic illustration of the retained anti-abrasion property. The micro-to-nanoscale structure (light grey particles) remains similar to the original morphology after abrasion, which retains the hierarchical roughness of the surface (in turn maintaining the superhydrophobicity).

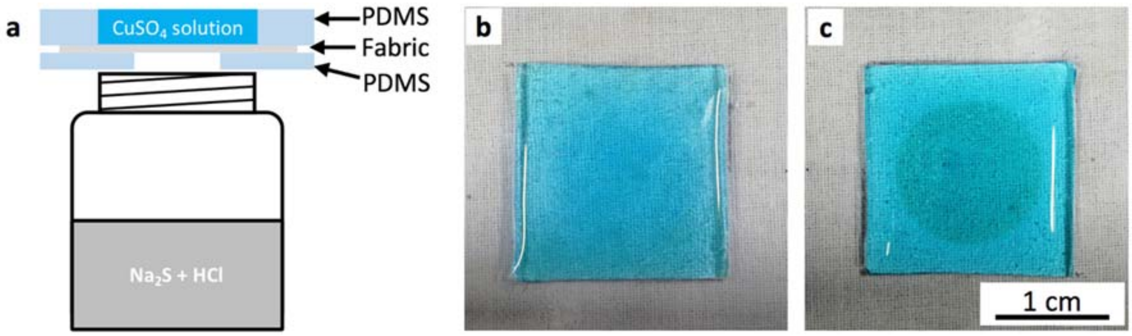

Supplementary Fig. 13 Gas permeation test of a treated cotton fabric piece. a Schematic view of the set-up. The fabric piece was clapped in between two pieces of PDMS (polydimethylsiloxane) plates. A bigger square was cut on the top layer of PDMS, a smaller circle was cut on the bottom layer of PDMS. In the bottle below, 50 mg Na_2S was added to the 0.1 M HCl solution; the generated H_2S gas would permeate through the fabric, react with $CuSO_4$ trapped at the top PDMS plate (hold by the superhydrophobic cotton fabric and the PDMS plate). **b** Top view of $CuSO_4$ solution before reaction. **c** Top view of the solution after reaction. A dark circle was formed after reaction, indicative of the formation of CuS (method adopted from Ref. 15). This result confirms that the superhydrophobic cotton fabric is gas permeable.

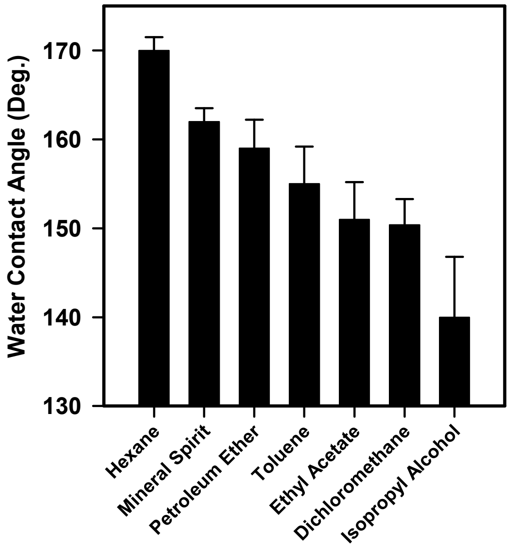

Supplementary Fig. 14 Comparison of different solvents for diluting the coating mixture. Water contact angles were measured on pieces of 1 × 3 cm^2 grade 1 filter paper that had been immersed for 2 h in the diluted solutions (of different solvents as listed). The error bars are derived from at least three independent experiments.

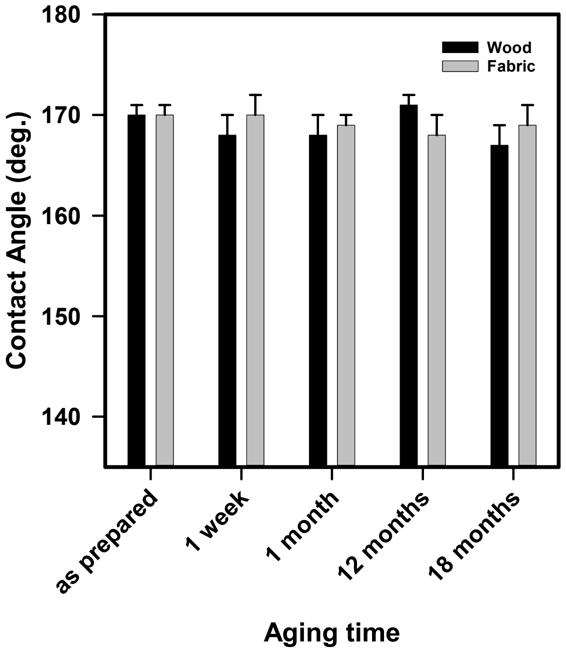

Supplementary Fig. 15 Aging effect of the coated plywood and cotton fabric samples. Upon storage under ambient conditions for 18 months, there are no apparent changes in either water contact angle or the sliding angle. The error bars show the standard deviations of at least independently prepared samples.

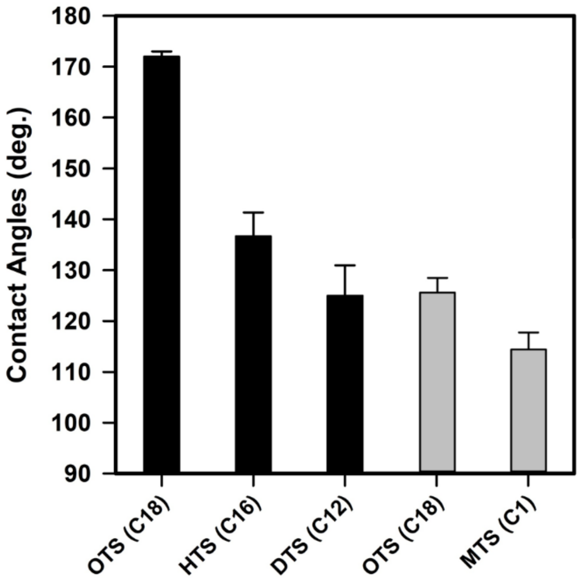

Supplementary Fig. 16 Comparison of different alkyltrichlorosilanes for the preparation of superhydrophobic coatings. The data for filter paper samples prepared with OTS (C18), HTS (C16), and DTS (C12) following the stoichiometric silanization protocol are shown as black bars. The error bars show the standard deviations of at least independently prepared samples. For comparison, the contact angles of samples modified with OTS and MTS via the conventional method are shown as grey bars; specifically, they were immersed in a diluted solution of the respective alkyltrichlorosilane (0.1 % v/v in hexane) for 10 min (Ref. 41). While HTS (C16) and DTS (C14) worked well with the stoichiometric silanization protocol, i.e., thus treated samples indeed become hydrophobic (137±5° and 125±6°) but not superhydrophobic. Through the conventional method, on the other hand, OTS and MTS modified samples exhibit even lower contact angles of 129±3° and 115±3°, respectively. Overall, none of the comparison groups, fabricated with either the conventional method or the present protocol, can reach the superior superhydrophobicity reported in this work (Figs. 1-2 of the main text).

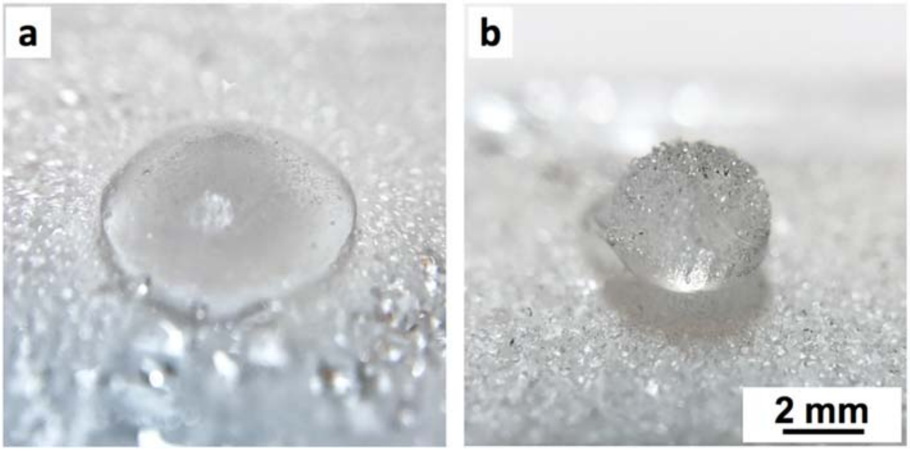

Supplementary Fig. 17 Anti-icing test of a treated glass slide. a An untreated glass slide. **b** A glass slide treated with the superhydrophobic coating. They were placed flat in a freezer (-20 ºC). 30 µL of water were carefully dropped on the surface of both slides, ensuring that the droplets stayed stationary. The samples were subsequently stored in the freezer overnight to allow the droplets to be frozen completely. Upon taking the samples out from the freezer, the anti-icing property was tested with a dual range force sensor (Vernier lab pro; Beaverton, OR) by measuring the adhesion force between the frozen droplet and the substrate surface. The diameter of droplet contact areas on two slides are 6.0 mm and 1.1 mm, respectively; the measured detachment force (normalized to the respective contact area) decreased ~30 %, from 895 ± 69 kN to 636 ± 36 kN (per m^2).

Table 1. Comparison of hydrophobic/superhydrophobic coatings using OTS as a precursor.

Ref.	Method	WCA	Function of OTS
This work	A small amount of water mixed with bulk OTS based on stoichiometry, followed by diluting and applying onto substrates via dipping or spraying.	>170°	Using OTS to create hierarchical micro/nanostructures with low surface tension in one step.
Ref. 25 Wong et al. *J. Chem. Edu.*	Glass slide was immersed in hexane solution of OTS for 1.5 h.	112° ± 3°	OTS reacts with hydroxyl groups on surface as well as neighboring molecules to form a SAM.
Ref. 27 Song et al. *Nano Res.*	Micro/nano morphological structures were created on silicon wafer with an aluminum-induced crystallization technique. Then OTS SAM was deposited on the modified silicon surface by immersing in an OTS/toluene solution.	155°	OTS self-assembly and crosslink on naturally hydrophilic micro/nanostructured surfaces to render superhydrophobicity.
Ref. 28 Zang et al. *Chem. Eng. J.*	Fiberglass cloth fiber was pre-"epoxidated" with an epoxy resin, followed with the deposition of amino-functionalized silica particles. It was then immersed into an OTS ethanol solution (60 °C for 2 h).	151° - 154°	Generate sufficient surface roughness by depositing amino-functionalized silica particle; and achieve low surface energy through reacting OTS with amine groups on amino-silica surface and with epoxy groups on epoxy resin.
Ref. 29 Yilbas et al. *Sci. Rep.*	Pre-cleaned silicon nanowires/nanowalls are first modified with OTS, followed by another coating of n-octadecane onto silicon wafer substrates.	165° ± 5°	The nanowire/nanowall structure contributes to the surface morphology, while the OTS modification lowers the surface energy.
Ref. 30 Ogihara et al. *Langmuir*	SiO_2 nanoparticles are first coated with OTS by immersing in an OTS/toluene solution. The coated nanoparticles are then added to an alcohol solution, which forms a suspension to be sprayed over other substrates.	153°	Nanoparticle suspension contributes to the hierarchical surface roughness, while OTS monolayer lowers the surface tension.
Ref. 31 Seeharaj et al. *J. Mater. Sci.*	SiO_2 nanoparticles are coated with chlorotrimethylsilane (CTMS) and OTS sequentially. The modified SiO_2 particles are ultrasonically dispersed in a polystyrene binder solution. The resulted mixture is then drop-coated on the substrate.	156° ± 1°	Chloro-groups from CTMS hydrolyzes to generate silanols, and then bond covalently with hydroxyl groups to form siloxanes on SiO_2. Next, OTS reacts with hydroxyl groups on SiO_2 particles to reduce the surface tension.
Ref. 32 Gan et al. Appl. *Surf.* Sci.	Wood samples are initially suspended in a solution of $FeSO_4$ and $CoCl_2$, followed by immersion in an OTS solution for 24 h.	>150°	The precipitation of $CoFe_2O_4$ nanoparticles on wood surface to introduce roughness, followed by OTS modification to lower the surface tension.

Ref. 33<br>Li et al.<br>*Chem. Eng. J.*	SiO_2 nanoparticles are modified with OTS first, followed by mixing with epoxy resin and polydimethylsiloxane in ethyl acetate solution. The suspension is then sprayed on the substrate.	159.5°	SiO_2 nanoparticles provides a "skeleton" that increases the surface roughness. OTS modification generates a low surface tension coating.
Ref. 34<br>Lv et al.<br>*RSC Adv.*	Cotton substrate is coated with a layer of SiO_2 particles by immersing into a TEOS solution, followed by dipping in an OTS/ethanol solution for 2 h.	151° ± 1.2°	SiO_2 nanoparticle coating introduces surface roughness, while the surface tension of cotton fibers is reduced via modification with TEOS and OTS.

Table 2. Comparison with commercially available spray coatings. The listed cost is the selling price per 100 mL spraying solution (in US$).

**Product**	**Solvent**	**Function Materials**	**Cost**	**Applicable products**	**WCA (deg.) on glass; fabric**
This work	mineral spirits	aggregated OTS	0.3*	glass, wood, paper, fabric, plastic, glass and metal	162±2; 165±2
Woods™ waterproof silicone spray	heptane, propane, stoddard solvent	not known	4.6	nylon, polyester tent, awnings, sleeping bags, snowsuits	108±2; 139±3
Grangers™ footwear repel	water	siloxane	4	footwear, suede, leather, fabric, nubuck, nylon, Sheepskin	101±3; 138±8
Woods™ instant waterproof spray	heptane, propane, mineral spirits	not known	3.7	tents, jackets, backpacks, footwear, headwear	116±2; 133±6
KIWI™ Protect-All™ leather & fabric footwear	mixture of hydrocarbons C7, butane, propane, N-butyl acetate, isobutane.	not known	6.4	shoes, bags, coats, outdoor equipment, suede, leather	108±3; 120±3
Nikwax TX. direct spray-on	water-based > 50%; acetic acid < 2%	not known	6.3	garments with knitted, wicking linings, bevy sacks, synthetic sleeping bags	94±4; 119±4
Scotchgard™ water shield heavy-duty water-repellent spray	hydrotreated light petroleum distillates, liquified, sweetened petroleum gases.	trimethylated silica	3.4	outdoor furniture fabrics, jackets, tents, hunting clothes, canvas shoes	82±7; 130±4
KIWI™ camp dry heavy-duty water repellent	mineral spirits or heavy naphtha, xylene, 1,2,4-trimethylbenzene	silicone	2.4	tents, tarps, boots, hunting apparel, outdoor gear, boat covers	100±2; 134±5
NeverWet®	mineral spirits	N/A	6.2	polyester, cotton, leather	110±5; 142±5

*Considering marginal profits, only material cost is calculated. The price of mineral spirits and OTS are1000 US$/ton and 1100 US$/ton, respectively (on average industrial price). Hence, the total material cost is 0.1 US$. We assume that the total cost is three times of the material expense.

Table 3. Comparison of representative superhydrophobic coatings with the present coating protocol.

Reference	Fabrication	WCA	Robustness
This work	Small amount of water first mixed with bulk amount of OTS based on stoichiometry. The mixture is later diluted and applied onto substrates by dipping or spraying.	>160°	Superhydrophobicity (>160°) on coated substrates resisted sand abrasion (4.5 kJ) for 10 min, water jetting (90 kJ) for 10 min, tissue wiping for 20 times, and moving 50 cm linearly on sandpaper under a load of 2.5 kPa.
Ref. 6<br>Deng et al.<br>*Science*	Substrate is initially covered with candle soot, followed by coating with tetraethoxysilane. The carbon soot core is later "burnt" through calcination, followed by a second coating of a semi-fluorinated silane via vapor deposition.	165° ± 1°	Coating resists sand abrasion from a height of 25 cm (2 m/s) for 5 min.
Ref. 8<br>Peng et al.<br>*Nat. Mater.*	Mixing polytetrafluoroethylene nanoparticles, perfluoropolyether, epoxy suspension, and fluorinated amine in acetone. The coating solution can be applied to substrates by spraying, brushing, or roll-coating.	~158°	The modified substrate is resistant to 30 cycles of tape peel test, 100 cycles of the standard Taber abrasion with loads of 150 g and 200 g, harsh chemical corrosion, and water jet at >35 m/s.
Ref. 9<br>Lu et al.<br>*Science*	Mixing two types of TiO_2 nanoparticles in an ethanol solution of perfluorooctyltriethoxysilane to make a "paint" before applying onto substrates along with adhesives (double-sided tape/spray adhesive) via spraying, dip-coating, or syringe extruding.	156° - 168°	Remained similar water repellency after finger-wipe, knife-scratch, and 40 cycles of sandpaper abrasion (10 cm abrasion per cycle).
Ref. 10<br>Pan et al.<br>*Nat. Mater.*	Spray-coating solution prepared by mixing 1H, 1H, 2H, 2H-perfluorohexyltrichlorosilane and *n*-butyl cyanoacrylate in dichloropentafluoropropane.	>150°	Superhydrophobic coating on polyester fabric endures scratch test, > 99 min sonication test, > 24 h washing test, > 100,000 drop impacting test, > 1,000 stretch–release cycles, > 100 self-healing cycles, and > 100 ice removal tests.
Ref. 43<br>Wang et al.<br>*Nature*	Surface modification includes a microstructure "armour" of micro-pyramid array and a nanostructure from candle soot or a commercially spray coating infill.	>150°	The superhydrophobicity withstood 1000 abrasion cycles with tape-peeling tests, Taber abrasion tests, scratch tests, and durability tests including thermal stability, chemical corrosion, water jet, high humidity environments.
Ref. 46<br>Gao et al.<br>*Adv. Mat.*	Pretreated-copper foam is dipped into a mixture of tetramethylethylenediamine, pyridine, and acetone, followed by adding hexakisbenzene dropwise. The resulted porous foam is coated with PDMS through vapor deposition.	157.6°	The contact angle dropped to 147.7° after 5 cycles of sandpaper abrasion (with 100 g weight on top, 20 cm abrasion distance each cycle).

Ref. 47<br>Li et al.<br>*Chem. Eng. J.*	The spray coating suspension is prepared by dissolving PDMS, polymercaptopropylmethlysiloxane, and 2- hydroxy-2-methyl-1-phenyl-1-propanone in hexane, followed by dispersing nano- and micro-silica into the solution.	160°	The contact angle remains above 155° after 18 cycles of abrasion on sandpaper (with 50 g weight on top, 20 cm abrasion distance each cycle).
Ref. 48<br>Zhang et al.<br>*Sci. Rep.*	Silica nanoparticles are dispersed in ethanol, followed by adding epoxy resin and dodecyltrimethoxysilane, and lastly a polyamide resin. Can be deposited via brush-coating, dip-coating, or spray-coating on substrates, allowing 24 h for curing.	154° ± 1.7°	Superhydrophobicity is maintained after 50 cycles of knife scratch and sandpaper abrasion. Per cycle of abrasion test involves moving substrate on sandpaper for 20 cm under a 100 g weight.
Ref. 49<br>Song et al.<br>*ACS Appl. Mater. Interfaces*	Drilling Al plate with laser and immersing in dilute HCl solution to create micro /nanometer-scale holes; the plate is then used as the mold to prepare superhydrophobic PDMS with pillars.	163°	Superhydrophobicity is not affected after scratching the sample with a steel ruler for 20 times.
Ref. 50<br>Zhang et al.<br>*Colloids Surf. Physicochem. Eng. Asp.*	A prepolymer, curing agent and polymerization inhibitor are mixed first, followed by adding to a silica nanoparticle solution in ethanol to form the spray-coating solution.	160°	Water contact angle >150° after 30 cycles of water drop impact (100 mL water released from 30 cm above the coated substrate).
Ref. 51<br>Ren et al.<br>*Surf. Coat. Technol.*	Perfluorooctyltriethoxysilane, ammonia, and tetraethylorthosilicate are mixed in ethanol to obtain a silica sol. Then an epoxy-resin-based adhesive is prepared by mixing epoxy resin, ethanol, tetraethylorthosilicate and 3-aminopropyltriethoxysilane, and subsequently mixed with the silica sol for coating sample surfaces.	164°	Tests with water droplet and sand impact test indicate good adhesion of the coating on the surface.
Ref. 52<br>Celik et al.<br>*Chem. Eng. J.*	Silica nanoparticles are coated with dodecyltrichlorosilane, and then mixed with carnauba wax in chloroform to complete the spray-coating solution.	175°	Superhydrophobicity is maintained after 1000 cycles of water spray, 45 min of water jet impact, and 180 cm of linear abrasion.
Ref. 53<br>Dong et al.<br>*Nanomaterials*	Employed electrical discharge machining to create uniformly distributed microscale craters and cracks on titanium surfaces.	162° ± 2°	Sandpaper abrasion test with 4.9 and 15 kPa load damages the superhydrophobicity after 660 and 420 cm abrasion distance, respectively.
