# Supplementary material for: Functional and versatile superhydrophobic coatings via stoichiometric silanization
Source: Nat Commun. 2021 Feb 12;12:982. doi: 10.1038/s41467-021-21219-y (PMC7881188; doi:10.1038/s41467-021-21219-y)
Supplement: Supplementary file 11 — Description of Additional Supplementary Files [file 41467_2021_21219_MOESM11_ESM.pdf]

Description of Additional Supplementary Files

Functional and versatile superhydrophobic coatings via stoichiometric silanization

Lishen Zhang, Alvin G. Zhou, Brigitta R. Sun, Kennedy S. Chen, and Hua-Zhong Yu*
Department of Chemistry, Simon Fraser University, Burnaby, British Columbia V5A 1S6, Canada
Correspondence to: hogan_yu@sfu.ca

Title: Supplementary Movie 1.
Description: A droplet (2 μL) of pure water pressed on and pulled back from the superhydrophobic glass slide with a needle, showing its superior non-sticky property.

Title: Supplementary Movie 2.
Description: Slow motion of a water droplet (50 μL, height 5 cm) impinging the superhydrophobic glass surface. The water droplet was bounced off from the substrate freely.

Title: Supplementary Movie 3.
Description: Slow motion of a water droplet (50 μL) sliding on a piece of treated glass slide (tilted to the left for 0.7°). The water droplet was bounced to another side instead of sticking on the surface.

Title: Supplementary Movie 4.
Description: Comparison of the prepared superhydrophobic filter paper (the coating is encapsulated with rhodamine B) and an untreated filter paper. Instead of adding water, 1.0 mM of rhodamine B solution was added. The slide was then placed under a hand-held UV lamp (254 nm). For the fluorescent sliding test, a pyranine solution was dropped on the slide (tilted at 0.7°). The untreated filter paper is tilted and tested under the same condition. While the superhydrophobicity is achieved, the treated paper retains the fluorescent property from the encapsulated rhodamine B.

Title: Supplementary Movie 5.

Description: Sliding angle test of the treated substrates (glass, paper, fabric, wood, aluminium, plastic). The superhydrophobic substrate was attached on a microscope glass slide with a double-sided tape; the glass slide was tilted from left to the right at 0.7°; a drop of water was pipetted on the surface at a distance of 5 cm.

Title: Supplementary Movie 6.

Description: Self-cleaning test of the treated substrates (glass and wood). A small piece of treated glass or wood was attached to a microscope glass slide with a double-sided tape. The glass slide was tilted from left to the right at 0.7°; MnO powders were spread on the surface, before dropping 50-μL water droplets.

Title: Supplementary Movie 7.

Description: Sand abrasion and water jetting tests of the treated filter paper. A triangular stage was fabricated with a 3D printer (JGAURORA, model A3S, HuaXun Technology Co. Ltd, Shenzhen, China; Printer Filament, AMZ3D), and the sample attached to a glass slide was placed on the stage (45° from the surface normal). The sand abrasion experiment was performed for 10 min at a height of 0.5 m (with a controlled flow rate of ~ 130 g/min), corresponding to an impinging energy of 4.5 J. The water jetting was also performed at a height of 0.5 m (with a controlled flow rate of ~260 g/min), corresponding an impinging energy of 9.0 J.

Title: Supplementary Movie 8.

Description: Sandpaper abrasion test of treated superhydrophobic filter paper (grade 1). A small piece of the treated superhydrophobic filter paper (1×2 cm^2) was affixed at the bottom of the weight (50 g). It was then pressed on a large piece of silicon carbide sandpaper (Grit No. 400) and moved for 50 cm.

Title: Supplementary Movie 9.

Description: A scale-up test of our coating method on cotton T-shirts. A cotton T-shirt was immersed in 1.5 L hexane solution of the coating mixture (5% v/v OTS/hexane), dried in the air upon removal. Tap water (dyed with green food color for better visualization) was poured into the T-shirt. The water was splashed off readily without sticking on the T-shirt.
